# The Therapeutic Potential of Targeting Substance P/NK-1R Interactions in Inflammatory CNS Disorders

**DOI:** 10.3389/fncel.2016.00296

**Published:** 2017-01-04

**Authors:** M. Brittany Johnson, Ada D. Young, Ian Marriott

**Affiliations:** Department of Biological Sciences, The University of North Carolina at CharlotteCharlotte, NC, USA

**Keywords:** substance P, neuroinflammation, neuropeptide, neurokinin-1 receptor, tachykinin, microglia, astrocytes

## Abstract

The inflammatory responses of resident central nervous system (CNS) cells are now known to play a critical role in the initiation and progression of an array of infectious and sterile neuroinflammatory disorders such as meningitis, encephalitis, Parkinson’s disease, Alzheimer’s disease and multiple sclerosis (MS). Regulating glial inflammatory responses in a timely manner is therefore critical in preserving normal CNS functions. The neuropeptide substance P is produced at high levels within the CNS and its selective receptor, the neurokinin 1 receptor (NK-1R), is abundantly expressed by neurons and is present on glial cell types including microglia and astrocytes. In addition to its functions as a neurotransmitter in the perception of pain and its essential role in gut motility, this tachykinin is widely recognized to exacerbate inflammation at peripheral sites including the skin, gastrointestinal tract and the lungs. Recently, a number of studies have identified a role for substance P and NK-1R interactions in neuroinflammation and described the ability of this neuropeptide to alter the immune functions of activated microglia and astrocytes. In this review article, we describe the expression of substance P and its receptor by resident CNS cells, and we discuss the ability of this neuropeptide to exacerbate the inflammatory responses of glia and immune cells that are recruited to the brain during neurodegenerative diseases. In addition, we discuss the available data indicating that the NK-1R-mediated augmentation of such responses appears to be detrimental during microbial infection and some sterile neurodegenerative disorders, and propose the repurposed use of NK-1R antagonists, of a type that are currently approved as anti-emetic and anti-anxiolytic agents, as an adjunct therapy to ameliorate the inflammatory CNS damage in these conditions.

Substance P is a member of the tachykinin family of neuropeptides that share common pharmacological properties and a conserved carboxyl-terminal sequence (Phe-X-Gly-Leu-Met-NH2, X hydrophobic or aromatic; Harrison and Geppetti, 2001). The carboxyl-terminal sequence is required for interaction with tachykinin receptors and cellular activation, while the distinct amino-terminal confers receptor subtype specificity (Gerard et al., 1991). The tachykinins are expressed broadly throughout the nervous and immune systems (Harrison and Geppetti, 2001; Satake and Kawada, 2006). Accordingly, these neuropeptides mediate a diverse range of physiological and pathological processes, and this has made them attractive targets for therapeutic intervention. The major mammalian tachykinins are substance P, neurokinin A, neurokinin B, neuropeptide K and neuropeptide gamma (Steinhoff et al., 2014). Of these, substance P is of particular interest as a target for the treatment of a variety of diseases due to its widespread distribution and its ability to induce inflammation, in contrast to other neuropeptides such as vasoactive intestinal peptide and pituitary adenylate cyclase-activating polypeptide that exert anti-inflammatory effects (Padua et al., 2016).

The inflammatory effects of substance P are mediated via interaction with its high affinity neurokinin-1 receptor (NK-1R; Pernow, 1983; Maggi, 1997), a seven transmembrane domain G-protein coupled receptor. While substance P preferentially interacts with, and signals via, NK-1R, at high concentrations it can also activate cells via neurokinin-2 and neurokinin-3 receptors (Regoli et al., 1994). Activation via NK-1R leads to cell-type dependent responses such as endothelial cell retraction and vascular smooth muscle dilation (Maggi, 1995; Holzer, 1998; van Hinsbergh and van Nieuw Amerongen, 2002). Perhaps more importantly, substance P can stimulate and/or modulate cytokine release by various cell types, and there is compelling evidence that this neuropeptide plays a critical role in modulating immune responses at peripheral sites such as the gastrointestinal and respiratory tracts where inflammation correlates with NK-1R activation (O’Connor et al., 2004). In the gastrointestinal tract, substance P regulates smooth muscle contractility, vascular permeability and immune functions (Pernow, 1983; Lördal et al., 1996; O’Connor et al., 2004). Elevated expression of substance P is associated with gastrointestinal diseases such inflammatory bowel disease, *Trichinella spiralis*-induced enteritis and *Clostridium difficile* enterocolitis (Koch et al., 1987; Mazumdar and Das, 1992; Swain et al., 1992; Agro and Stanisz, 1993; Bernstein et al., 1993) and substance P levels correlate with symptom severity in cryptosporidiosis (Robinson et al., 2003). The link between substance P and damaging inflammation in the gut is further supported by the demonstration that NK-1R blockade abrogates intestinal inflammation associated with *Clostridium difficile* toxin A and *Trichinella spiralis*-induced enteritis (Swain et al., 1992; Agro and Stanisz, 1993; Kataeva et al., 1994; Castagliuolo et al., 1998). Interestingly, the presence of proinflammatory cytokines can further induce the level of expression of NK-1R by colonic epithelial cells suggesting that positive feedback loops may exist to potentiate the pro-inflammatory actions of substance P in the gastrointestinal tract (Goode et al., 2003). Similarly, in the respiratory tract, interactions between substance P and NK-1R have been shown to augment inflammatory processes where this neuropeptide can increase immune cell infiltration and the release of cytokines that contribute to disease pathology in asthma and respiratory syncytial virus infection (Nadel, 1991; King et al., 2001).

As we discuss in the present review, substance P is the most abundant tachykinin in the brain (Pernow, 1983; Severini et al., 2002) and NK-1R can be expressed by non-neuronal cells of the central nervous system (CNS), such as astrocytes and microglia, that have important immune functions (Michel et al., 1986; Beaujouan et al., 1991; Marriott and Wilkin, 1993; Lai et al., 2000; Rasley et al., 2002b). Similar to reports in the gastrointestinal and respiratory tract, substance P has been shown to augment inflammation in the CNS (Lee et al., 1994; Rasley et al., 2004b; Chauhan et al., 2008). This ability, and the availability of centrally acting NK-1R inhibitors that are approved for use in human subjects, raises the intriguing possibility that the targeting of substance P/NK-1R interactions could be useful as an adjunctive therapy for the treatment of neuroinflammatory disorders.

## Localization of Substance P and NK-1R within the CNS

Tachykinins were originally named for their ability to stimulate contraction of intestinal muscle in the seminal work by V Euler and Gaddum (1931) in which “preparation P”, later renamed substance P, isolated from horse intestine and brain induced isolated rabbit jejunum contraction. It is now appreciated that substance P and its receptor are found in high quantities throughout the CNS. Substance P immunoreactivity was observed in the CNS as early as the mid-1970s (Hökfelt et al., 1975; Cuello and Kanazawa, 1978; Ljungdahl et al., 1978a,b; Cuello et al., 1979) and neuroanatomical analysis of substance P distribution in the rat CNS revealed the highest levels in the substantia nigra and the medial amygdaloid nucleus of the brain, and in the superficial dorsal horn of the spinal cord (Ribeiro-da-Silva and Hökfelt, 2000). The presence of substance P within the primary sensory neurons of the brainstem, cranial nerve nuclei, and spinal cord dorsal horn (Cuello and Kanazawa, 1978; Cuello et al., 1979, 1993; Douglas and Leeman, 2011) is consistent with its role as a sensory neurotransmitter important for the perception of pain (Douglas and Leeman, 2011).

However, substance P is found in many other regions of the brain including the hippocampus, cortex, basal ganglia and hypothalamus (Ebner and Singewald, 2006), and can produce effects throughout the CNS. Furthermore, this tachykinin is also broadly distributed throughout the peripheral nervous system and enteric nervous system, and can be expressed by cells of the immune system. Specifically, substance P can be expressed by peripheral leukocytes including lymphocytes and monocytes/macrophages (Bost et al., 1992; Bost, 1995; Ho et al., 1997; Lai et al., 1998; Chernova et al., 2009).

NK-1R is a G-protein coupled receptor and has the highest affinity for substance P over other tachykinin receptors and is found as two isoforms; a full-length form (407 amino acids) and a truncated form (311 amino acids; Fong et al., 1992; Baker et al., 2003). The truncated form of NK-1R binds substance P with 10-fold lower affinity than the long form (Douglas and Leeman, 2011). Substance P binding to full-length NK-1R stimulates phosphorylation of the C-terminus via G-protein receptor kinases and protein kinase C to activate members of the mitogen-activated protein kinase (MAPK) cascade, including extracellular signal-regulated kinases 1 and 2, and p38 MAPK (as reviewed in Steinhoff et al., 2014). Importantly, in immune cells such as macrophages, activation of MAPK cascades precipitates activation of NF-kB, the master regulator of immune mediator production (Sun et al., 2008). Hence, through activation of NF-κB, substance P can augment inflammation by stimulating the production of proinflammatory cytokines (Bost, 2004a). In contrast, the truncated NK-1R lacks the majority of the C-terminus and is not phosphorylated following substance P binding. Accordingly, signaling via this truncated form does not result in NF-kB activation, and fails to elicit cellular effects including desensitization, endocytosis, endosomal signaling changes and production of inflammatory chemokines such as IL-8 (DeFea et al., 2000; Lai et al., 2008). However, the truncated NK-1R form may have biological functions beyond serving as a decoy receptor, and has been suggested to provide or augment a persistent growth stimulus to cancer cells (Gillespie et al., 2011).

Substance P mediates numerous and cell-type specific effects throughout the body due to the broad expression of NK-1R in many cell types and tissues. Like substance P, NK-1R expression is widely distributed throughout the CNS as determined by autoradiography, *in situ* hybridization and immunohistochemical approaches (Mantyh et al., 1984, 1989; Nakaya et al., 1994; Caberlotto et al., 2003). NK-1R mRNA is found in the olfactory bulb, cerebral cortex, medulla oblongata and spinal cord in mice (Andoh et al., 1996). Initial studies of human brain revealed high levels of NK-1R expression in the locus coeruleus and ventral striatum, moderate expression in the cerebral cortex, hippocampus and amygdaloid nuclei, and only low levels in the cerebellum and thalamus (Caberlotto et al., 2003). Further studies, using positron emission tomographic analysis of healthy human males using a high-affinity ^18^F labeled-NK-1R antagonist, suggested that the highest levels of cellular NK-1R expression normally occur in the caudate and the putamen, regions of the brain in close proximity to the amygdaloid nucleus (Hietala et al., 2005). The constitutive expression and anatomical location of this neuropeptide and its receptor in the limbic system of the healthy brain suggest that substance P/NK-1R interactions play an integral role in complex CNS processing, and the neurotransmitter/neuromodulatory functions of this tachykinin have recently been reviewed elsewhere (Garcia-Recio and Gascón, 2015).

It is noteworthy that there has been considerable recent interest in defining the location of NK-1R expressing cells with the recognition that substance P may not act as a classical neurotransmitter. Rather, there are anatomical mismatches between substance P release sites and responsive cells (Liu et al., 1994; Nakaya et al., 1994). Liu et al. (1994) determined that many substance P receptors had non-synaptic localizations within the rat CNS. The ability of substance P to diffuse and act upon distant cells suggests that this neuropeptide could have endocrine characteristics within the CNS and beyond. The relevance of this is underscored by the fact that numerous non-neuronal cell types, such as smooth muscle cells, fibroblasts, keratinocytes and endothelial cells, functionally express NK-1R. Importantly, glial cells with immune functions and leukocytes recruited to the CNS also express NK-1R, either constitutively or following activation (Schaffer et al., 1998), and so may be susceptible to the pro-inflammatory actions of this neuropeptide. However, it should be noted that the cellular expression of the truncated NK-1R form can also be dynamic (Vilisaar et al., 2015), and few studies have distinguished between the full length and truncated form of this receptor.

## Substance P Promotes Leukocyte Recruitment and Activation

While the CNS is extensively protected by the blood-brain barrier, inflammation within the brain leads to the infiltration of peripheral macrophages, dendritic cells, T cells and other immune responders (Hickey, 1999; Whitney et al., 2009), and it is well recognized that there is extensive crosstalk between the nervous system and the immune system. Trauma and infections of the CNS are characterized by high levels of inflammatory mediators such as cytokines (Ziebell and Morganti-Kossmann, 2010), and immunomodulatory neuropeptides including substance P can affect the release of cytokines by immune cells (Lee et al., 1994; Ho et al., 1996). In turn, inflammatory cytokines can regulate the release of substance P and expression of NK-1R by leukocytes, further sensitizing these cells to the effects of substance P in a positive feedback loop-like manner (Marriott and Bost, 2000; Blum et al., 2001, 2008; Weinstock et al., 2003a).

The interaction of substance P with NK-1R triggers neurogenic inflammation and directly augments inflammatory processes in the lung, gut, skin and other peripheral organs (Steinhoff et al., 2014). Neurogenic inflammation is characterized by vasodilation and increased vascular permeability leading to increased immune cell infiltration (Brown and Neher, 2010). Substance P promotes inflammation in at least three ways as recently discussed by Corrigan et al. (2016). First, substance P elicits vasodilation and increases vascular permeability. Second, this neuropeptide can facilitate leukocyte extravasation by inducing the expression of adhesion molecules necessary for immune cell adherence to endothelial cells, and by subsequently promoting their migration to the site of injury or infection. Third, substance P can act directly on resident and/or infiltrating cells to augment their immune functions.

Early reports identified the expression of substance P and/or NK-1R by peripheral human lymphocytes and monocytes/macrophages (Bost et al., 1992; Ho et al., 1997; Lai et al., 1998). Since these studies, natural killer cells, dendritic cells, mast cells and neutrophils have been added to the list and have been shown to either express NK-1R or respond to substance P in an NK-1R-dependent manner (Marriott and Bost, 2001a; O’Connor et al., 2004). Accordingly, during an immune challenge, substance P produced by immune and non-immune cell types can function as a potent chemoattractant and promote the inflammatory responses of a diverse range of cells including macrophages, dendritic cells, lymphocytes and eosinophils (O’Connor et al., 2004). However, it should be noted that the mechanisms underlying the actions of substance P on eosinophils are more controversial as its effects have been reported to occur in a receptor-independent manner, either via the N-terminus of substance P or via interactions with epithelial cells (Kroegel et al., 1990).

Substance P has been shown to also stimulate or augment the production of inflammatory cytokines by monocytes/macrophages, T lymphocytes and mast cells, and can promote the release of inflammatory mediator-containing granules of neutrophils, mast cells and eosinophils (Bill et al., 1979; Shanahan et al., 1985; Lotz et al., 1988; Serra et al., 1988; Kroegel et al., 1990; Calvo et al., 1992; Ho et al., 1996; Marriott and Bost, 2001b). In addition, this neuropeptide can also inhibit the production of the immunosuppressive cytokine TGF-β1 by activated macrophages (Marriott and Bost, 1998) providing another mechanism by which this tachykinin can foster an inflammatory environment. Furthermore, substance P has been reported to stimulate T-lymphocyte proliferation and natural killer cell activity, and substance P may also serve as a B-lymphocyte differentiation cofactor and augment immunoglobulin secretion (Croitoru et al., 1990; Pascual and Bost, 1990; Pascual et al., 1991a,b, 1992; Bost and Pascual, 1992; Covas et al., 1994). As such, these studies indicate that this neuropeptide has the potential to exacerbate both acute and chronic inflammatory immune responses associated with the recruitment of immune cells to the CNS.

Due to the dynamic crosstalk between the nervous system and the immune system, it is perhaps not surprising that the expression of both substance P and its receptor are regulated by immune responses. The potent inflammatory mediator IL-12, the related cytokine IL-23 and IL-18, can all induce substance P production and NK-1R expression by murine macrophages and T-lymphocytes (Blum et al., 2001, 2008; Weinstock et al., 2003a; Arsenescu et al., 2005). In addition, a recent study has provided evidence for the reciprocal positive regulation of IL-23 and IL-17 pathways and NK-1R expression in human mononuclear cells (Vilisaar et al., 2015). The ability of IL-12/IL-23 cytokines and IL-18 to induce NK-1R expression can be explained due to the ability of both of these cytokines to elicit cellular effects via NF-kB activation (Weinstock et al., 2003a), and the fact that the promoter region of the NK-1R gene contains a binding site for this transcription factor (Takahashi et al., 1992). Similarly, other pro-inflammatory cytokines including IFN-γ and TNF-α can elevate NK-1R expression in macrophages, while this effect can also be achieved with the signature T-helper type 2 cytokine, IL-4 (Marriott and Bost, 2000).

In contrast, cytokines with anti-inflammatory functions, such as IL-10 and TGF-β, have been reported to reduce the expression of substance P and NK-1R in immune cells (Weinstock et al., 2003b; Blum et al., 2008). However, caution should be taken in designating the effects of pleiotropic cytokines such as TGF-β. While TGF-β is generally considered to be immunosuppressive, the presence of this cytokine in combination with inflammatory cytokines, such as IL-6, promotes the formation of proinflammatory TH17 T-cells (Bettelli et al., 2006). Furthermore, there is evidence that this cytokine can down-regulate NK-1R internalization by T-cells in a mouse model of inflammatory bowel disease, and can enhance signal transduction pathways used by effector T-cells to augment inflammatory cytokine production (Beinborn et al., 2010).

## Substance P Promotes the Inflammatory Immune Responses of Resident CNS Cells

The brain has typically been characterized as a victim organ of infiltrating leukocytes, but it is increasingly appreciated that resident glial cells play an essential role in the initiation and progression of immune responses within the CNS. While, early studies reported substance P immunoreactivity in cells and tissues isolated from the CNS (Michel et al., 1986; Kostyk et al., 1989), it is now recognized that non-neuronal CNS cells can express both substance P and its receptor, either constitutively or following exposure to inflammatory/damaging stimuli, much like peripheral immune cells. Cortical and white matter astrocytes express substance P and NK-1R (Michel et al., 1986; Torrens et al., 1989; Beaujouan et al., 1991; Marriott and Wilkin, 1993), and this receptor has also been detected in human spinal cord astrocytes (Palma et al., 1997). Like leukocytes, the expression of substance P and its receptor by astrocytes is influenced by inflammatory mediators. For example, levels of NK-1R expressed by a human astrocytic cell line and rat primary astrocytes are elevated in response to the potent inflammatory cytokine IL-1β, consistent with previous observations that substance P binding sites are highly expressed following neuronal injury (Guo et al., 2004).

While the expression of substance P receptors by neurons, cerebral endothelial cells, and astrocytes is well established, the expression of authentic substance P receptors on microglia is more controversial. Luber-Narod et al. (1994) reported a lack of detectable binding sites for radiolabeled substance P on microglia, either at rest or following challenge with bacterial lipopolysaccharide. Similarly, another study failed to detect *in vivo* NK-1R expression by rat microglia following ischemic brain injury as determined by *in situ* hybridization and immunohistochemical staining approaches (Stumm et al., 2001). In contrast, our own studies indicate that neonatal mouse microglia express mRNA encoding NK-1R and we have confirmed the presence of this receptor protein in this cell type by Western blot analysis and flow cytometry (Rasley et al., 2002b). Likewise, NK-1R has been specifically detected in human fetal brain microglia and rat spinal microglia (Lai et al., 2000; Bradesi et al., 2009).

Importantly, the interaction of substance P with NK-1R appears to promote inflammatory immune responses by glia in a similar manner to its effects on peripheral leukocytes. Substance P activates the key inflammatory regulator NF-κB in human astrocytoma cells (Lieb et al., 1997), and can elicit the production of a number of inflammatory cytokines, including IL-6, IL-8 and granulocyte macrophage colony-stimulating factor (GM-CSF), and reactive oxygen intermediates by these cells and primary human embryonic and spinal astrocytes (Lieb et al., 1997; Palma et al., 1997; Palma and Manzini, 1998). Similarly, we have shown that this neuropeptide induces the nuclear translocation of the RelA subunit of NF-kB (Rasley et al., 2002b) in primary murine microglia, while Zhu et al. (2014) have recently shown that exposure to substance P, in combination with histamine, results in the production of reactive oxygen species and the release of IL-6 and TNF-α by these cells. Since microglia and astrocytes are susceptible to the pro-inflammatory effects of substance P, this neuropeptide appears to be capable of exacerbating the immune responses of both resident CNS cells and recruited leukocytes in CNS damage or disease.

## Evidence for the Involvement of Substance P in CNS Disorders

Given that both resident glia and infiltrating leukocytes can express substance P and its receptor, either constitutively or following exposure to immune mediators, and that substance P is present at high levels throughout the CNS, it is perhaps not surprising that this neuropeptide has been implicated in the pathogenesis of a number of inflammatory CNS disorders.

It is well known that CNS trauma and infection are accompanied by widespread inflammation in the brain and resident glial cells, including astrocytes and microglia, play a key role in the initiation and propagation of this response (Corrigan et al., 2016). Although protected extensively by the blood-brain barrier, neuroinflammation leads to infiltration of peripheral macrophages, dendritic cells, T cells, and other immune responders (Hickey, 1999; Whitney et al., 2009). Activated glial cells secrete a plethora of pro-inflammatory mediators including PGE_2_ (Rasley et al., 2002a, 2004b), IL-12 (Rasley et al., 2004a), IL-6, and TNF-α (Chauhan et al., 2009, 2010) resulting in the disruption of the blood-brain barrier thereby facilitating cellular infiltration (Hickey, 1999; Lossinsky and Shivers, 2004; Taupin, 2008; Whitney et al., 2009). Accordingly, the ability of substance P to induce vasodilation in the brain, promote the recruitment of immune cells, and augment the inflammatory responses of both infiltrating and resident cells, can contribute to the development of devastating conditions such as meningitis and encephalitis following infection (Maggi, 1995; O’Connor et al., 2004; Corrigan et al., 2016).

A compelling body of evidence now suggests that interactions between substance P and NK-1R exacerbate CNS inflammation during parasitic, viral and bacterial infections. Neurocysticercosis is a parasitic infection of the CNS caused by *Taenia solium* that results in seizures in patients due to the granulomatous host immune response to this pathogen. Analysis of biopsies from human neurocysticercosis patients has shown the presence of substance P positive cells adjacent to degenerating worms, and this neuropeptide is expressed in granulomas in murine models of neurocysticerosis (Robinson et al., 2002). Interestingly, administration of substance P into the rodent hippocampus can induce seizures, and levels of substance P correlate with granuloma formation and seizure activity in animal models of CNS helminth infection. Furthermore, substance P/NK-1R interactions are required for cytokine responses and seizures associated with neurocysticerosis granulomas (Garza et al., 2008, 2010). Human African trypanosomiasis also affects the CNS and substance P has similarly been implicated in the neuropathology of this protozoan parasite. Inhibition of substance P/NK-1R interactions using the non-peptide NK-1R antagonist RP-67580 reduces the inflammatory response and the reactive astrogliosis associated with *Trypanosoma brucei brucei* infection of the CNS (Kennedy et al., 1997), further supporting a role for substance P/NK-1R interactions in neuroinflammation associated with eukaryotic parasite CNS infections.

Bacterial infections of the CNS are serious and often intractable conditions affecting the meninges and the brain parenchyma. During bacterial meningitis, increased cellularity and reactive astrogliosis are hallmarks of an active immune response within the CNS. Such responses indicate activation of resident astrocytes and the accumulation of cells at the site of bacterial challenge, either due to proliferation of glial cells including microglia, or the recruitment of peripheral leukocytes. Furthermore, the recruitment and/or activation of resident glial cells and infiltrating leukocytes are associated with high CNS levels of inflammatory mediators resulting in neurological dysfunction. Using mice deficient in the expression of NK-1R or the prophylactic administration of the NK-1R antagonist L703,606, we have demonstrated that substance P interactions with NK-1R are required for the increases in blood-brain barrier permeability, astrogliosis, increased CNS cellularity, and elevated numbers of microglia/macrophages associated with infection with the clinically relevant bacterial CNS pathogens *Streptococcus pneumoniae*, *Neisseria meningitidis* and *Borrelia burgdorferi* (Chauhan et al., 2008, 2011). Furthermore, in these studies we have shown that substance P/NK-1R interactions are involved in bacterially-induced demyelination and behavioral changes following infection with these disparate bacterial species. Importantly, the decreases in bacterial-induced CNS disease severity seen with NK-1R inhibition or deficiency were associated with diminished CNS production of key inflammatory mediators including IL-6 and TNF-α, and the prevention of infection-induced decreases in the level of the anti-inflammatory cytokine IL-10.

Interestingly, bacterial infection in these mouse models is associated with elevated levels of NK-1R expression in the brain and, specifically, by microglia and astrocytes (Chauhan et al., 2011). Such increases are particularly important since we have shown that substance P can significantly increase bacteria induced IL-6 production by microglia (Chauhan et al., 2008). Furthermore, this neuropeptide can augment microglial expression of the pro-inflammatory enzyme cyclooxygenase-2 and its product prostaglandin E2, and elevate expression of the prostanoid receptors EP2 and EP4 to further potentiate inflammation (Rasley et al., 2004b). Together, these animal studies indicate a pivotal role for substance P in the initiation and progression of damaging neuroinflammation following bacterial infection of the CNS.

Similarly, intense CNS inflammation can occur following viral infection and a relationship between substance P and human immunodeficiency virus (HIV) infection has been suggested. Elevated levels of substance P have been observed in the serum of HIV infected patients and simian immunodeficiency virus-infected rhesus macaques (Douglas et al., 2001, 2008a,b; Lai et al., 2001; Vinet-Oliphant et al., 2010). Viral levels correlate with the amount of substance P released by immune cells and it has been suggested that this neuropeptide may facilitate viral replication by increasing the expression of receptors including CCR5 that are required for HIV infection of host cells (Bost, 2004a; Manak et al., 2010). In the CNS, human fetal brain cells have been shown to express NK-1R and interactions between this receptor and substance P appear to exacerbate HIV-1 infection (Schwartz et al., 2013).

Finally, substance P interactions with NK-1R have been associated with CNS disorders that are generally associated with sterile inflammation such as Alzheimer’s disease, Parkinson’s disease, multiple system atrophy (MSA) and multiple sclerosis (MS). However, it seems that this influence may center on the neuroprotective effects of this neurokinin. Decreased levels of substance P have been reported in animal models of CNS motor disorders and in the brain tissue of postmortem Parkinson’s disease patients (Chen et al., 2004). Similarly, in Alzheimer’s disease, reduced levels of substance P are observed in the cortical regions of postmortem brain tissues and in patient cerebrospinal fluid (Quigley and Kowall, 1991; Kowall et al., 1993; Waters and Davis, 1997; Raffa, 1998). Furthermore, in MSA and Parkinson’s disease patients there is severe depletion of NK-1R expressing neurons in the ventrolateral medulla (Benarroch et al., 2003). But even this apparently benign role may have a darker side as substance P immunoreactive astrocytes have been identified in MS plaques (Kostyk et al., 1989), and a requirement for substance P/NK-1R interactions has been reported for the maintenance of chronic inflammation in experimental autoimmune encephalomyelitis (EAE) mouse models of MS (Reinke et al., 2006). Such a detrimental role is also supported by genome-wide linkage studies identifying the TAC1 gene encoding the substance P precursor protein as a possible MS susceptibility gene (Vandenbroeck et al., 2002; Cunningham et al., 2007).

Together, the available data indicate that interactions between substance P and NK-1R dictate the inflammatory response observed in diverse CNS infections or disorders. Additionally, the amount of substance P released by neurons and immune cells, along with the number of available receptors, determines the level of neuroinflammation, as greater release of substance P permits diffusion of this neuropeptide to more distal binding sites broadening its effect (Abbadie et al., 1996; Doyle and Hunt, 1999; Mantyh, 2002).

## Substance P as a Double-Edged Sword in CNS Immune Responses

An active immune response can be protective as well as detrimental in the CNS. While infiltrating leukocytes and resident glia play a role in limiting infection and trauma-associated damage, immune responses generated by these events may prove detrimental if not controlled and limited at the appropriate time (Douglas and Leeman, 2011). Indeed, multiple neurodegenerative disorders are associated with exacerbated immune responses by resident and recruited cells in the brain. Therefore, a delicate balance needs to be struck in order to limit the generation of damaging inflammation within the CNS.

For some bacterial and viral infections, substance P has been demonstrated to be necessary for clearance. For example, murine gammaherpesvirus 68 has been demonstrated to increase the expression of substance P and its receptor in mucosal and lymphoid organs. Importantly, NK-1R deficient mice show reduced CTL responses and IL-12 secretion, resulting in increased viral burden compared to wild type animals (Elsawa et al., 2003). Similarly, substance P/NK-1R interactions are required for resistance to Salmonella infection as demonstrated by the advanced salmonellosis and reduced survival rates in infected mice treated with an NK-1R antagonist (Kincy-Cain and Bost, 1996, 1997). Here again, substance P promotes IL-12 expression and this leads to IFN-γ production that drives the cell-mediated immunity required to clear this intracellular bacterial pathogen.

In contrast, this neuropeptide appears to contribute to disease pathology for some infectious agents. For example, substance P increases the bronchoconstriction and damaging cardiac inflammation following infection with respiratory syncytial virus and encephalomyocarditis virus, respectively (Bost, 2004b; Robinson et al., 2009). Likewise, substance P contributes to the severity of inflammation associated with *Trypanosoma brucei* infection and inflammation and granuloma size in a mouse model of *Taenia solium* cysticercosis (Kennedy et al., 1997; Garza et al., 2008, 2010).

As we discussed earlier, our data suggest that substance P similarly exacerbates damaging inflammation within the CNS to disparate bacterial pathogens. We determined that the absence of substance P/NK-1R interactions in substance P receptor deficient mice or prophylactic pharmacological NK-1R inhibition in wild type animals significantly reduces bacteria-induced neuroinflammation and resultant CNS damage (Chauhan et al., 2008, 2011). NK-1R null mice and mice treated with an NK-1R antagonist showed reduced inflammatory and maintained immunosuppressive cytokine production, as well as decreased astrogliosis, cellularity and demyelination following intracerebral administration of the Gram negative bacterial pathogens *N. meningiditis* and *B. burgdorferi*, or the Gram positive bacterium *S. pneumoniae* (Chauhan et al., 2008, 2011). These animal studies therefore indicate that substance P/NK-1R interactions are essential for the progression of damaging inflammation following bacterial CNS infection.

Targeting NK-1R also has the potential to ameliorate CNS disorders that are thought to involve sterile inflammation given the presence of substance P immunoreactive glia in MS plaques (Kostyk et al., 1989) and the reported ability of the non-peptide NK-1R antagonist SR140333 to attenuate chronic inflammation associated with mouse models of MS (Reinke et al., 2006). However, the benefit of inhibiting substance P/NK-1R interactions in other neurological disorders, including Alzheimer’s disease and Parkinson’s disease, is more equivocal due to the reported neuroprotective properties of this neuropeptide and/or its ability to stimulate non-amyloidogenic amyloid precursor protein processing (as discussed in Chen et al., 2004; Severini et al., 2016).

As such, the available data suggests that substance P contributes to neuroprotection during some degenerative CNS disorders and to beneficial cell-mediated host responses against viruses and intracellular bacteria facilitating pathogen clearance. Inhibiting substance P/NK-1R interactions in these conditions would likely compromise the protective effects of this neuropeptide and exacerbate disease severity. In contrast, NK-1R-mediated augmentation of glial and recruited leukocyte immune responses appears to be detrimental during extracellular bacterial and parasite infections of the CNS, and perhaps MS, by exacerbating neuroinflammation and neurological damage (as summarized in Table 1). Based on these studies, it appears that targeting substance P/NK-1R interactions might be a promising strategy to ameliorate the inflammatory CNS damage associated with such infectious agents and neurological disorders.

**Table 1:** **Models of neuroinflammation in which NK-1R antagonists and/or genetic deficiency have shown reduced disease severity**.

CNS disorder	Model	Organism	Antagonist	Knockout	Effect	Reference
Neurocysticersosis	*T. crassiceps*	Mouse		NK-1R Substance P	Lower granuloma volume. Less IL-1β, IL-6, TNF-α.	Garza et al. (2010)
African trypanosomiasis	*T. brucei* + diminazene	Mouse	RP-67580		Reduced astrogliosis, clinical score, cellularity.	Kennedy et al. (1997)
Bacterial meningitis	*S. pneumoniae* *N. meningiditis* *B. burgdorferi*	Mouse	L703,606	NK-1R	Reduced BBB permeability, astrogliosis, microgliosis, cellularity, demyelination, IL-6, TNF-α. More IL-10.	Chauhan et al. (2008) Chauhan et al. (2011)
Multiple sclerosis	EAE	Mouse	SR 14033	NK-1R	Greater mobility. Decreased MOG specific T-cells.	Reinke et al. (2006)
			CP-96,345		Less clinical and histological signs.	Nessler et al. (2006)

## The Therapeutic Potential of NK-1R Antagonists in Inflammatory CNS Disorders

The involvement of tachykinins in a wide range of pathological processes has made them an attractive target for therapeutic intervention, and the pharmaceutical industry has initiated the development of additional NK-1R antagonists (as discussed in Quartara and Altamura, 2006; Quartara et al., 2009). Many of these NK-1R antagonists have reached phase II and III clinical trials with one, aprepitant, currently approved by the United States Food and Drug Administration (Quartara and Altamura, 2006; Quartara et al., 2009; Di Fabio et al., 2013). The non-peptide NK-1R antagonist CP-96,345 has been reported to downregulate constitutive substance P mRNA expression in human mononuclear cells (Lai et al., 2002) and the ability of such NK-1R antagonists to treat a range of gastrointestinal, respiratory and urogenital, and sensory disorders has been explored. However, the utility of next generation NK-1R antagonists in the treatment of CNS conditions including nausea, addiction, pain, and depression, has been of particular interest due to their ability to cross the blood-brain barrier.

The first NK-1R antagonists were developed in the early 1990s, but most were not effective as analgesics and anti-depressants as they could not efficiently cross the blood-brain barrier. An exception to this rule was LY303870, which was shown to block rodent licking behavior in the late stages of persistent nociceptive activation with inhibition of *ex vivo* substance P binding to both peripheral and central NK-1R (Iyengar et al., 1997). Subsequently, an analysis of the pharmacokinetics of the non-peptide NK-1R antagonist ezlopitant in dogs revealed the presence of this compound and its two pharmacologically active metabolite compounds in cerebrospinal fluid following intravenous or oral administration, indicating its ability to cross the blood brain barrier (Reed-Hagen et al., 1999). Of the latest generation of NK-1R antagonists, aprepitant has been shown to cross the blood-brain barrier after oral administration using human positron emission tomography to demonstrate its ability to occupy NK-1R within the brain in an oral dose and plasma concentration dependent manner (Bergström et al., 2004). Similarly, the NK-1R antagonist casopitant, when radioactively labeled, has been demonstrated to be rapidly absorbed into the bloodstream and can subsequently be found within the brain (Ruhlmann and Herrstedt, 2009). This antagonist has completed phase II and III clinical trials and has similar success to aprepitant in the treatment of chemotherapy-induced nausea and vomiting (Ruhlmann and Herrstedt, 2009), but this drug has not yet received United States Food and Drug Administration approval.

The ability of NK-1R antagonists to cross the blood-brain barrier means that these agents have the potential for use in the treatment in wide range of CNS disorders. Indeed, aprepitant and its pro-drug fosaprepitant are currently employed as post-chemotherapy anti-emetic agents (Aapro et al., 2015). Likewise, casopitant has completed phase II and phase III trials and has similar success to aprepitant in the treatment of chemotherapy-induced nausea and vomiting (Ruhlmann and Herrstedt, 2009). In addition to use as an anti-emetic, there has been promising research that aprepitant and other NK-1R antagonists may have efficacy against other CNS disorders including depression. The NK-1R antagonist MK-869 has been shown to effectively suppress depressive behavior in guinea pigs, and both MK-869 and casopitant successfully completed phase II clinical trials to treat depression (Kramer et al., 1998; Ebner and Singewald, 2006; Di Fabio et al., 2013). However, it must be noted that MK-869 failed in a phase III trial to treat major depressive disorder (Keller et al., 2006).

There are also hopes that NK-1R antagonists may be valuable for the treatment of other CNS disorders as diverse as schizophrenia, panic attacks, Parkinson’s disease, and MS. This hope stems from the promising effects of NK-1R antagonists capable of crossing the blood-brain barrier in animal models of such disorders (Table 1). For example, the NK-1R antagonist SR140333 has shown efficacy as a co-therapy with other anti-inflammatory agents in ameliorating myelin oligodendrocyte glycoprotein-induced EAE, a mouse model of MS (Reinke et al., 2006). Similarly, CP 96345, has also been demonstrated to reduce EAE severity, and this effect was associated with stabilization of the blood brain barrier as well as reduced T-helper type 1 immunity (Nessler et al., 2006).

Importantly, given a potential role for substance P/NK-1R interactions in damaging inflammatory responses within the CNS following infection, there is considerable interest in targeting this receptor to limit neuroinflammation and neurological sequelae associated with infectious agents (Table 1). For example, since direct hippocampal substance P administration elicits seizures in mice and an NK-1R antagonist has been shown to prevent seizure activity in a rodent model of helminth brain infection (Robinson et al., 2012), it is possible that blocking the actions of this neuropeptide may prove useful in neurocysticercosis patients. Furthermore, our own studies have shown that pharmacological targeting of NK-1R with the antagonist L703,606 can not only prevent the development of damaging inflammation due to streptococcal CNS infection when administered prophylactically, but can also reverse infection-associated gliosis and demyelination when delivered therapeutically without increasing CNS bacterial burden (Chauhan et al., 2011).

While further studies are clearly needed to define the specific mechanisms underlying the ability of substance P to augment CNS inflammation and its role in pathogen clearance, the available data raise the intriguing possibility that currently approved NK-1R antagonists, such as aprepitant, could be repurposed for use as a co-therapy to limit neuroinflammatory damage associated with infectious agents and certain neurodegenerative conditions. The use of such agents might have considerable advantages over other anti-inflammatory agents such as corticosteroids, non-steroidal anti-inflammatory drugs, prostaglandin inhibitors, and P2X7 antagonists, by limiting excessive inflammation without broadly attenuating host immune responses that may be required to resolve infection. Continued research on the ability of NK-1R antagonists to alleviate these health conditions may therefore yield new treatment options for patients with CNS infections, and perhaps other inflammatory neurological disorders.

## Concluding Remarks

Infectious and sterile CNS disorders are often associated with overwhelming and damaging inflammation due to the immune responses of resident CNS cells and infiltrating leukocytes. Currently, neuroinflammatory diseases such as meningitis are treated with corticosteroids in combination with antibacterial, antiviral, or antifungal agents (Aberdein and Singer, 2006; Hoffman and Weber, 2009). Corticosteroids have both anti-inflammatory and immunosuppressive properties and treatment with these agents has been shown to reduce the risk of hearing loss and mortality due to meningitis. However, the immunosuppressive properties of corticosteroids may interfere with the ability of the body to clear infections, especially if the antibiotics used are not effective or antiviral medications are not available (Fitch and van de Beek, 2008). Furthermore, corticosteroid treatment can lead to adverse effects including the development of ulcers, myopathy, and bone loss, or central effects including sleep disorders and mood swings. As such, there is a current need for new treatment options to limit neuroinflammation associated with infection of the CNS and neurodegenerative conditions.

**Figure 1:**
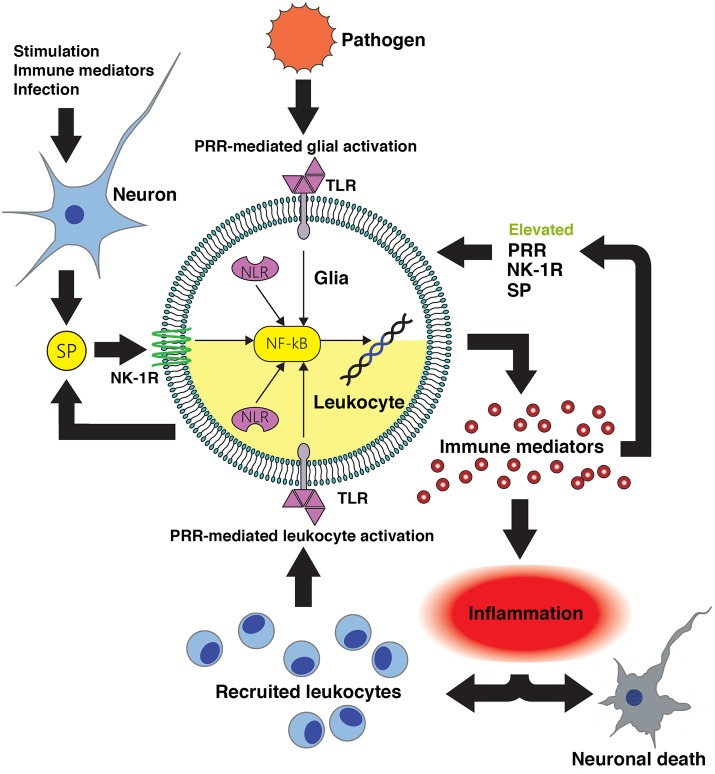
**Substance P-mediated exacerbation of neuroinflammatory damage following central nervous system (CNS) infection.** Conserved microbial motifs are recognized by Toll-like receptor (TLR) and NOD-like receptors (NLR) pattern recognition receptors (PRR) expressed by perivascular macrophages, microglia and astrocytes leading to NF-kB activation and inflammatory and/or neurotoxic mediator production. Substance P (SP) released by neurons and perhaps activated glia acts on neurokinin-1 receptor (NK-1R) bearing CNS cells to augment NF-kB activation or function to enhance glial responses. Local inflammation promotes the recruitment of leukocytes to the site of infection that will, in turn, recognize microbial components and produce more pro-inflammatory and neurotoxic mediators. Infiltrating leukocytes such as monocytes/macrophages, dendritic cells and lymphocytes express NK-1R and so are similarly susceptible to the pro-inflammatory actions of substance P. Importantly, inflammatory cytokines can also augment the expression of PRR and NK-1R by glia and leukocytes, and can elevate local production of substance P. This positive feedback loop would be anticipated to increase the sensitivity of host cells to pathogen components and sensitize leukocytes and glia to this neuropeptide, thereby exacerbating inflammatory damage. Intervention with pharmaceutical NK-1R inhibitors capable of penetrating the blood-brain barrier would prevent substance P mediated exacerbation of glial and leukocyte inflammatory responses, and interrupt such a feedback loop.

Substance P is produced at high levels in the CNS, and its target receptor NK-1R is expressed by resident CNS cells including microglial and astrocytes, and by immune cells that can infiltrate the CNS such as macrophages and lymphocytes. Importantly, this tachykinin functions both as a neurotransmitter and an immunomodulator, and substance P is recognized to exacerbate inflammatory responses at peripheral sites including the skin, lung and gastrointestinal and urogenital tracts. In infectious and sterile CNS conditions associated with severe neuroinflammation, substance P/NK-1R interactions appear to augment inflammation by increasing the release of inflammatory mediators while concomitantly decreasing the production of anti-inflammatory cytokines from microglia and astrocytes (as summarized in Figure 1) thereby exacerbating neuronal damage (Chauhan et al., 2008, 2011). Our own studies indicate that therapeutic intervention with NK-1R antagonists can limit neuroinflammation, reactive gliosis and demyelination in a mouse model of streptococcal meningitis (Chauhan et al., 2011) while other groups have shown efficacy of these agents in animal models of MS (Nessler et al., 2006; Reinke et al., 2006). This data therefore supports the use of NK-1R receptor antagonists in the treatment of such neuroinflammatory disorders.

The latest generation of NK-1R antagonists can be delivered orally and can readily cross the blood-brain barrier. Importantly, these antagonists exert central effects and the NK-1R antagonist, aprepitant, is approved for use as an anti-emetic agent in patients receiving chemotherapy. As such, the repurposing of currently available NK-1R antagonists may yield alternative co-therapy options for CNS inflammation associated with extracellular bacteria and parasites, and perhaps MS, with more specificity and reduced adverse effects.

## Author Contributions

All authors have contributed significantly to the preparation of this review article.

## Funding

This work is supported by the NINDS (National Institutes of Health) research grant R01 NS050325 awarded to IM.

## Conflict of Interest Statement

The authors declare that the research was conducted in the absence of any commercial or financial relationships that could be construed as a potential conflict of interest.
